# Editorial: The Role of Reactive Oxygen Species in Protective Immunity

**DOI:** 10.3389/fimmu.2021.832946

**Published:** 2022-01-25

**Authors:** Denis Martinvalet, Michael Walch

**Affiliations:** ^1^ Department of Biomedical Sciences, University of Padua, Padova, Italy; ^2^ Veneto Institute of Molecular Medicine, Padova, Italy; ^3^ Faculty of Science and Medicine, Department of Oncology, Microbiology and Immunology, Anatomy Unit, University of Fribourg, Fribourg, Switzerland

**Keywords:** reactive oxygen species (ROS), protective immunity, innate immunity, adaptive immunity, immunoregulation

## Introduction

Reactive oxygen species (ROS) result from the partial reduction of oxygen, and encompass both radical [superoxide anion (O_2_^•-^), hydroxyl radical (^•^OH), and nitric oxide (NO)] and non-radical [hydrogen peroxide (H_2_O_2_), hypochlorous acid (HOCl), and peroxynitrite (ONOO^-^)] species having different half-life and reactivity. Classically, ROS have most often been considered harmful as they are common determinants of many cell death pathways including apoptosis, necrosis/necroptosis, ferroptosis, pyroptosis and autophagic cell death (1–8). This obvious ROS toxicity is certainly beneficial in oxidative killing of engulfed microbial pathogens in specialized immune cells, such as neutrophils (9) or macrophages (10). However, the simplistic view that ROS can only be harmful have been revisited. Indeed, ROS are essential to many physiological processes. ROS contribute to inflammation, vasoconstriction, signal transduction, cell migration, differentiation and proliferation (11–14). Moreover, H_2_O_2_ can modulate genes expression by redox based-epigenetic modification (15–17) and at the transcriptional level by activating redox responsive transcription factors such as AP-1, NRF2, CREB, HSF1, HIF-1, TP53, NF-κB, NOTCH, SP1, SCREB-1 and FOXO family (18–26). H_2_O_2_ also acts at the posttranscriptional level to control gene expression by regulating both cap-dependent and cap-independent translation (27–29). Because of their rather pleiotropic actions, ROS are critically important for cell biology, organ function, and system physiology including that of the immune system. In this Research Topic, we aimed at taking a close look at how ROS contribute to protective immunity.

## ROS in Antimicrobial Immune Defense

ROS have been implicated in many aspects of the immune response to pathogens. ROS can damage biomolecules by oxidizing iron-sulfur clusters in a variety of enzymes leading to metabolic defects and release of iron. Free iron can react with hydrogen peroxide (H_2_O_2_) to give rise to aggressive hydroxyl radicals that can damage any biomolecule, including DNA. Therefore, ROS are essential for pathogens killing by phagocytic cells as illustrated by chronic granulomatous disease (CGD), an inherited disorder of the NADPH oxidase characterized by recurrent and severe bacterial and fungal infections (30). Reciprocally, microbes developed many strategies to counteract ROS- dependent host defense mechanism as discuss by Sen and Imlay. Indeed, microbes sense the H_2_O_2_ *via* OxyR or PerR transcription factors, or use the Grx3/Yap1 system to initiate enzymes that reduce cytoplasmic H_2_O_2_ concentrations, decrease the intracellular iron pools, and repair the H_2_O_2_-mediated damage and suit their particular environmental niches.

In addition to the ability of ROS to directly kill bacteria in the phagosome by oxidative damage to essential biomolecules (31), they can also trigger pathogen defense of phagocytes by various non-oxidative means, such as autophagy, receptor signaling, extracellular traps formation and instructing lymphocyte responses. Interestingly, ROS critically orchestrate the inflammatory process by regulating cytokine production during infection. Adding to this line of research, Hatinguais et al. demonstrated that sublethal ROS were essential to proinflammatory cytokine expression in the context of macrophages infected with swollen *Aspergillus fumigatus conidia*. The source of these ROS was identified in mitochondria by reverse electron flow, although NADPH oxidase 2 seemed to play a regulatory role in this proinflammatory pathway. A study by Buvelot et al. showed that sublethal doses of hydrogen peroxide trigger a coordinated and massive downregulation of genes involved in pyrimidine metabolism in *Staphylococcus aureus*, leading to reduced growth of extracellular bacteria and an increased sensitivity to added H_2_O_2_. Strikingly, as opposed to extracellular bacteria, intracellular pathogens were less affected, which could be a long-term survival strategy by allowing colonization through intracellular survival, while decreasing the risk of killing the host through dampened extracellular growth.

The induction of caspase-mediated host cell death can critically contribute to the elimination of intracellular pathogens *via* the destruction of their niche and *via* the induction of efferocytosis (31–33). However, many obligate intracellular pathogens have evolved mechanisms to inhibit programed cells death. Lavergne et al. discuss how this limitation is dealt with by the cytotoxic lymphocyte proteases, the granzymes. In addition to triggering host cell death, they also exert various non-cytolytic antimicrobial activities by directly degrading vital microbial proteins or hijacked host proteins crucial for the replication or survival of the pathogens. The granzymes also target microbial virulent factors. Interestingly, many mechanisms applied by the granzymes in this context rely on the induction of reactive oxygen species, either by promoting host cell apoptosis or by inhibition of pathogen growth. Whether the ROS involved in pathogen killing, cell death induction and the regulation of host cell metabolism originated from the same source is not fully elucidated and will need further study.

## Regulation of Immune Cells by ROS

Beside the well-established ROS involvement in pathogen elimination, the wide range of the regulatory capacity of these reactive molecules is more and more revealed. Karmakar et al. discuss the regulatory mechanism downstream of the binding of Siglecs to sialic acid decorated receptor on immune cells. The Siglecs are a family of sialic-acid-binding immunoglobulin-like lectins believed to promote intercellular interactions and regulate the functions of innate and adaptive immune cells (34). Siglecs modulate immune activation and can promote or inhibit ROS generation under different contexts. dssSiglecs can bind sialoglycans present on the same cell (cis-interactions) or extracellular ligands present on neighboring cells or secretory glycoproteins (trans-interactions). Siglec-sialoglycan binding is weak and transient (22). The interaction between Siglecs with multivalent ligands leads to Siglec clustering, which increases the strength of Siglec-ligand binding and initiates cellular signaling.

ROS have also been proposed to be the common determinant of the inflammasome activation critical in the inflammatory process which is the determinant for an efficient immune response (35–38). The RNA-binding protein tristetraprolin (TTP) is an anti-inflammatory factor that prompts the mRNA decay of target mRNAs and is involved in inflammatory diseases such as rheumatoid arthritis (RA). lv et al. have shown that protein phosphatase 2A (PP2A)-mediated dephosphorylation regulates TTP to activate its mRNA-degrading function. Interfering with TTP expression or agonist of PP2A modulate monosodium urate (MSU) crystal-induced the expression of inflammation-related genes and NLRP3 inflammasome activation in a mitochondrial ROS-dependent manner suggesting that targeting TTP expression or function may provide a potential therapeutic strategy for inflammation caused by MSU crystals.

These studies demonstrate that ROS exert highly pleotropic functions in immunity. Therefore, it is essential to identify the specific intracellular sources of ROS and how they influence cellular processes in both physiological and pathological means, and how they impact on metabolic processes and inflammatory signaling as discussed by Canton et al. in the context of macrophages. Moreover, ROS are also required for full activation of lymphocytes as well as for the regulation of autoimmunity. Indeed, Bassoy et al. highlight the contribution of ROS in lymphocyte biology and stress their contribution in adaptive immunity with direct impact on the outcome of the antitumoral immune response as a consequence of the redox state in the tumor microenvironment (TME). Cali et al. showed the detrimental tolerogenic role of tumor-infiltrating myeloid cells (TIMs) actively dismantling effective immunity against cancer. TIMs inhibit T cell functions and promote tumor progression by multiple mechanism including the potentiation of the oxidative/nitrosative stress within the TME. They demonstrated that nitrosylation of granulocyte monocyte stimulating factor (GM-CSF) nourishes the expansion of this highly immunosuppressive myeloid subsets in tumor-bearing hosts.

The contribution of the ROS in adaptive immunity was further developed by Mortimer et al. who focus on the emerging role of NOX2-derived ROS in the development and maintenance of adaptive immunity and the effects of excess ROS in systemic disease. To this regard, Chávez and Tse discussed the impact of mitochondrial-derived ROS and immunometabolism reprogramming in autoreactive T cell differentiation. Dysfunctional mitochondria have been involved in oxidative stress associated with many T cell-mediated autoimmune diseases. This agrees with the ability of mitochondrial-derived ROS to also contributed to T cell fate and function. Therefore, targeted manipulation of glycolysis and mitochondrial derived ROS could contribute to the elimination of autoreactive T cells while promoting immunosuppressive CD4 T regulatory (Treg). This targeted manipulation would have the advantage of avoiding global immunosuppression and preserving physiological immune response.

Finally, given the essential role of ROS for basic physiological functions as well as their contribution to pathophysiological situation, their therapeutic manipulation becomes an attractive strategy, although a non-trivial one. Dumas and Knaus discuss essential consideration for effective Redox medicinal approaches.

## Conclusion

This special Research Topic covers the extremely wide span of ROS function in immune defense and regulation and, with that, also the urgent need for further study to exploit these pleotropic molecules for therapy approaches. ROS are not only essential for antimicrobial defense but also exert a variety of crucial regulatory functions, reaching from the modulation of transcriptional programs to mediating differentiation fate. On the other hand, oxidative stress can contribute to various pathologies, including neuronal degenerative diseases, autoimmunity as well as cancer. Therefore, the potential therapeutic intervention in ROS biology will need extensive temporal and spatial fine tuning.

## Author Contributions

DM and MW wrote the manuscript. All authors contributed to the article and approved the submitted version.

## Funding

Work from Martinvalet’s lab was supported by grants from UNIPD SID 2018 and ERC starting grant ERC-2010-StG_20091118. Work from the Walch’s lab was supported by the Swiss National Science Foundation (SNSF grant # 310030_169928), the Novartis Foundation for Medical-Biological Research and the Vontobel-Foundation (all to MW).

## Conflict of Interest

The authors declare that the research was conducted in the absence of any commercial or financial relationships that could be construed as a potential conflict of interest.

## Publisher’s Note

All claims expressed in this article are solely those of the authors and do not necessarily represent those of their affiliated organizations, or those of the publisher, the editors and the reviewers. Any product that may be evaluated in this article, or claim that may be made by its manufacturer, is not guaranteed or endorsed by the publisher.
